# Synergistic Interaction of Clusters of Iron Oxide Nanoparticles and Reduced Graphene Oxide for High Supercapacitor Performance

**DOI:** 10.3390/nano12152695

**Published:** 2022-08-05

**Authors:** Amir Elsaidy, Julia N. Majcherkiewicz, Begoña Puértolas, Verónica Salgueiriño, Xosé Ramón Nóvoa, Miguel A. Correa-Duarte

**Affiliations:** 1 CINBIO, Universidade de Vigo, 36310 Vigo, Spain; 2 Departamento de Física Aplicada, Universidade de Vigo, 36310 Vigo, Spain; 3 CINTECX, ENCOMAT Group, EEI, Universidade de Vigo, 36310 Vigo, Spain

**Keywords:** clusters of iron oxide nanoparticles, hybrid nanocomposite, reduced graphene oxide, supercapacitors

## Abstract

Supercapacitors have been recognized as one of the more promising energy storage devices, with great potential use in portable electronics and hybrid vehicles. In this study, a composite made of clusters of iron oxide (Fe_3_O_4_-γFe_2_O_3_) nanoparticles and reduced graphene oxide (rGO) has been developed through a simple one-step solvothermal synthesis method for a high-performance supercapacitor electrode. Electrochemical assessment via cyclic voltammetry, galvanostatic charge–discharge experiments, and electrochemical impedance spectroscopy (EIS) revealed that the Fe_3_O_4_-γFe_2_O_3_/rGO nanocomposite showed much higher specific capacitance than either rGO or bare clusters of Fe_3_O_4_-γFe_2_O_3_ nanoparticles. In particular, specific capacitance values of 100 F g^−1^, 250 F g^−1^, and 528 F g^−1^ were obtained for the clusters of iron oxide nanoparticles, rGO, and the hybrid nanostructure, respectively. The enhancement of the electrochemical performance of the composite material may be attributed to the synergistic interaction between the layers of graphene oxide and the clusters of iron oxide nanoparticles. The intimate contact between the two phases eliminates the interface, thus enabling facile electron transport, which is key to attaining high specific capacitance and, consequently, enhanced charge–discharge time. Performance evaluation in consecutive cycles has demonstrated that the composite material retains 110% of its initial capacitance after 3000 cycles, making it a promising candidate for supercapacitors.

## 1. Introduction

Supercapacitors have attracted considerable attention as energy storage devices for various applications, such as portable electronics, pulse power technologies, and hybrid vehicles, owing to their unique properties in terms of high power density, fast charge–discharge capability, excellent cycling stability, reduced weight and size, easy operation, and higher energy efficiency over batteries and fuel cells [1,2,3,4]. Supercapacitors have been classified into two categories, i.e., electrical double-layer capacitors (EDLCs) and pseudocapacitors. The first class of electrochemical supercapacitors supply electrical energy via the accumulation of charge at the electrode/electrolyte interface, whilst the pseudocapacitors can supply energy through charge transfer redox reactions occurring at the electrode surface [5,6,7]. The latter class, in which the electrode is mostly based on transition metal oxides, exhibits better capacitive behavior [8]. Among the various metal oxides, iron-based oxides and hydroxides, e.g., single oxides (Fe_2_O_3_, Fe_3_O_4_, FeOOH, etc.) and binary metal oxides (MFe_2_O_4_ (M = Ni, Co, Sn, Mn, Cu), etc.) have attracted increasing attention due to the multiple oxidation states of iron, their rich redox chemistry, their low toxicity, and their abundance on Earth, which make them suitable for commercial applications [9,10,11]. In particular, Fe_2_O_3_- and Fe_3_O_4_-based nanostructured materials for supercapacitors have been widely investigated [9]. The applicability of bare Fe_2_O_3_ is limited as its poor electrical conductivity (10^−14^ S cm^−1^) and insufficient ionic diffusion rate result in a specific capacitance still far below the high theoretical expected value [12,13]. To overcome these issues, several approaches, including the construction of Fe_2_O_3_ composite electrodes using conductive supports [14,15], oxygen vacancy-induced Fe_2_O_3_ electrodes [13,16], or the fabrication of Fe_2_O_3_-Fe_3_O_4_ hybrid metal oxide composites, have been reported [17,18]. The relatively good electrical conductivity of Fe_3_O_4_ (10^2^–10^3^ S cm^−1^) was explored in combination with the poorly conductive Fe_2_O_3_ phase by Chen et al. [19], who reported a α-Fe_2_O_3_/Fe_3_O_4_ heterostructured nanoparticle, and by Tang et al. [17], who fabricated hierarchical Fe_2_O_3_@Fe_3_O_4_ core–shell nanorod arrays, in which Fe_3_O_4_ was used as a conductive support. The synergistic effect between Fe_2_O_3_ and Fe_3_O_4_ phases results in an electrochemical performance superior to that exhibited by the individual components. However, despite their initial promising capacitive behavior, they generally undergo deactivation upon use in consecutive cycles [20,21]. This poor capacitive performance and bad cycling stability are mainly due to the agglomeration of Fe_3_O_4_ during the charging/discharging process, which results in a low surface area and structural pulverization.

An effective strategy to prevent the agglomeration of the Fe_3_O_4_ materials is to integrate them with highly conductive carbon-based materials. For instance, Fe_3_O_4_ electrodes incorporating activated carbon [22,23], acetylene black [9], graphene [24,25], graphite [26], and carbon nanotubes [11,27] have been reported as efficient materials for supercapacitors, in which the carbon-based component can work as a conductive channel for electron diffusion. In comparison to carbon nanotubes and activated carbon, graphene-based materials possess enhanced mechanical properties, higher electrical conductivity, and a larger surface area [9], which makes them suitable candidates for supercapacitor applications. Composites based on magnetite (Fe_3_O_4_) nanoparticles in combination with rGO [28], N-doped graphene [29] or graphene sheets [30] have been extensively reported, but the number of studies approaching the introduction of clusters of iron oxide nanoparticles decorating the graphene sheets is relatively scarce [31].

In this study, we report the synthesis of a composite based on clusters of iron oxide nanoparticles and rGO through a simple one-step solvothermal synthesis, and its electrochemical behavior, using the clusters of iron oxide nanoparticles and/or the rGO as reference materials. The synthesized samples have been fully characterized, and their performance has been evaluated by cyclic voltammetry, galvanostatic charge–discharge experiments, electrochemical impedance spectroscopy (EIS), and long-term cyclic stability.

## 2. Materials and Methods

### 2.1. Chemicals

Iron (III) chloride hexahydrate (>99%), sodium acetate (>99%), poly(ethylene glycol) (MW 6000, PEG 6000), graphene oxide (GO) powder (flakes), ethanol (synthesis grade), 5 wt. % Nafion^TM^ perfluorinated resin solution, potassium hydroxide (>85%), sodium acrylate (97%), diethylene glycol (DEG, >99%), and ethylene glycol (EG, >99%) were purchased from Sigma-Aldrich (Madrid, Spain), and were used as received.

### 2.2. Materials Synthesis

The synthesis of clusters of iron oxide nanoparticles was conducted via the solvothermal method using a previously reported synthetic protocol [32]. Typically, 2.5 mmol (0.678 g) of iron(III) chloride hexahydrate were dissolved in 20 mL of ethylene glycol, and mechanically stirred to the formation of a clear solution, followed by the addition of sodium acetate (1.8 g) and polyethylene glycol (PEG 6000) (8.5 wt. %). The mixture was stirred vigorously for 30 min and then sealed in a stainless steel autoclave (100 mL). The autoclave was heated up to 185 °C (heating rate = 5 °C min^−1^) and kept at this temperature for 8 h under stirring (1500 rpm). Finally, the mixture was allowed to cool down to room temperature and the black powder was collected using a magnet. The resulting nanostructure was washed several times with Milli-Q water, and dried at 60 °C overnight.

Reduced graphene oxide–iron oxide (Fe_3_O_4_-γFe_2_O_3_/rGO) hybrid nanocomposites were synthesized following a previously reported method with some modifications [33]. Briefly, 0.05 g of GO sheets were dispersed in 20 mL of an EG:DEG mixture (EG:DEG = 1:19 vol.%) under ultrasonication. Subsequently, 1 g of sodium acrylate, 0.678 g of iron chloride hexahydrate, and 1.8 g of sodium acetate were added into the suspension, followed by the addition of 1.7 g (8.5 wt. %) of PEG. The resulting mixture was vigorously stirred until a homogeneous dispersion was obtained followed by the solvothermal treatment in a stainless steel autoclave using the same conditions as described for the preparation of clusters of iron oxide nanoparticles. In this case, sodium acrylate acts as a stabilizer preventing the aggregation and sodium acetate assists in the reduction of FeCl_3_ to Fe_3_O_4_ by altering the alkalinity [33]. The as-prepared composite was collected using a magnet and washed several times with ethanol and water, and then dried at 60 °C overnight. A control sample (Fe_3_O_4_-γFe_2_O_3_/rGO-C) was prepared using 0.015 g of GO sheets and following the same experimental procedure. Similarly, pure rGO was synthesized and used as a reference.

### 2.3. Materials Characterization

X-ray diffraction (XRD) patterns were collected using a Siemens D-5000 powder X-ray diffractometer operated in Bragg Brentano geometry using Ni-filtered Cu Kα radiation (*λ* = 0.1541 nm). Data were recorded in the 2*θ* range 10–80° with an angular step size of 0.026° and a counting time of 1 s per step. The collected data were refined using the Le Bail method by means of the software Rietica [34,35]. Raman spectra were collected from powder samples onto a glass slide as substrate, with a Renishaw in Via Reflex Raman microscope (Renishaw, Gloucestershire, UK). Experiments were conducted at room temperature using 532 and 633 nm excitation wavelengths. Field emission scanning electron microscopy (FESEM) was conducted on a JEOL JSM-6700 F. Samples for transmission electron microscopy (TEM) studies were prepared by dropping a diluted suspension of the samples onto ultra-thin carbon-coated copper grids. Imaging was performed on a JEOL JEM 1010 instrument operated at 100 kV and equipped with a CCD camera (JEOL, Tokyo, Japan).

### 2.4. Electrochemical Tests

The electrochemical measurements were conducted in a standard three-electrode cell configuration using a PalmSens4^®^ potentiostat. The electrochemical cell was filled with 25 mL of a 3 M KOH solution prepared with Milli-Q water. A Pt wire spiral and a Ag/AgCl (3.0 M KCl) were used as the counter and reference electrode, respectively. The working electrodes were prepared as described elsewhere [36]. In total, 0.3 mg of the sample was dispersed in 24 µL of ethanol and then 6 µL of the prepared solution was drop-cast onto a carbon gas diffusion layer (1 cm × 1 cm; Sigracet 39BB) four times followed by the addition of 3 µL of 5 wt. % perfluorinated Nafion^TM^ resin solution, which acts as a binder. Cyclic voltammetries, galvanostatic charge–discharge scanning experiments at different scan rates, and cyclic stability tests at a 50 mV s^−1^ scan rate (3000 cycles) were conducted in the −0.1 to +0.35 V potential window. Electrochemical impedance spectroscopy (EIS) data were obtained at the respective open circuit potential, from 50 kHz down to 0.1 Hz, taking 10 frequencies per decade, with ±5 mV sinusoidal voltage excitation.

## 3. Results and Discussion

### 3.1. Sample Characterization

**Figure 1:**
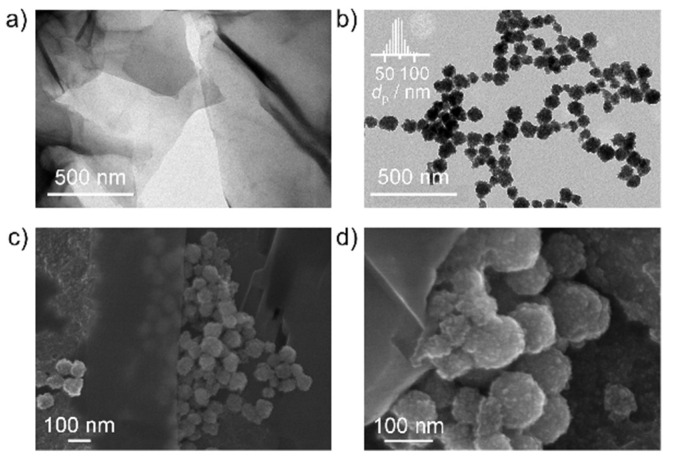
TEM micrographs of the (**a**) rGO sheets and (**b**) clusters of Fe_3_O_4_-γFe_2_O_3_ nanoparticles. Panels (**c**,**d**) correspond to SEM images of the Fe_3_O_4_-γFe_2_O_3_/rGO hybrid.

The clusters herein studied were obtained through a solvothermal method. The formation mechanism proceeds via a two-stage growth process, with nucleation of the primary nanocrystals followed by uniform aggregation into larger secondary structures [37], whose size is primarily determined by the polyethylene glycol concentration in the reaction medium. Figure 1 and Figure S1 (see Supplementary Materials) include the TEM and SEM images of these clusters of iron oxide nanoparticles and of the iron oxide/reduced graphene oxide hybrid. TEM micrographs confirm the formation of iron oxide nanoparticles grouped in clusters of controlled size as corroborated by the size distribution analysis, which evidenced the formation of the clusters with an average diameter of 70 ± 19 nm (Gaussian fit, inset in Figure 1b). Complementarily, SEM images reveal the formation of the individual clusters of iron oxide nanoparticles in between the rGO layers.

**Figure 2:**
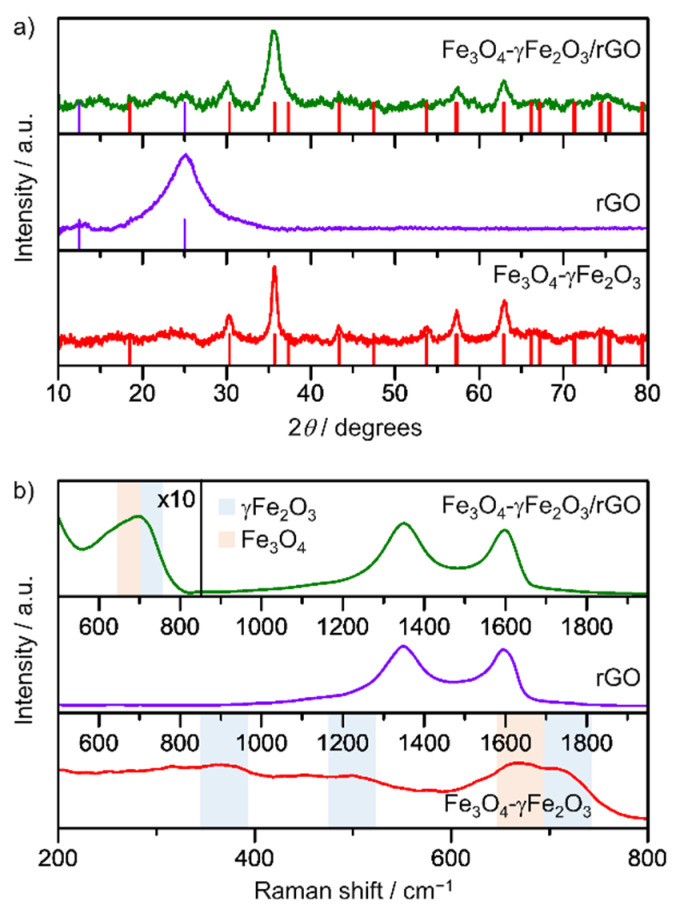
(**a**) X-ray diffractograms and (**b**) Raman spectra of the rGO sheets, clusters of Fe_3_O_4_-γFe_2_O_3_ nanoparticles, and Fe_3_O_4_-γFe_2_O_3_/rGO hybrid. The boxed region in (**b**) is scaled by a factor of 10.

The XRD results of the samples are compiled in Figure 2a. The diffractogram of rGO displays a broad peak at 2*θ* = 25° that corresponds to the (002) plane. Clusters of Fe_3_O_4_-γFe_2_O_3_ nanoparticles are composed of crystallites of small sizes whose crystalline structure corresponds to either magnetite (Fe_3_O_4_), maghemite (γ-Fe_2_O_3_), or a mixture of them, as both iron oxides present the same spinel structure (on which Fe^3+^ or Fe^2+^ cations or Fe^3+^ cations and vacancies are arranged, respectively). Similarly, these diffraction peaks along with the broad band between 20° and 30° associated with rGO are detected in the hybrid structure. Indeed, the intensity of the latter is quite low, which might be due to its highly disordered structure because of oxidation and the low content. In order to shed light on the nature of the iron oxide species in the samples, we completed the structural analysis using Raman spectroscopy (Figure 2b). This technique enables us to register the different vibrations of the crystalline lattice due to different cationic arrangements [38,39], and can therefore differentiate the two magnetic iron oxide phases. The Raman spectrum of the clusters of Fe_3_O_4_/γ-Fe_2_O_3_ nanoparticles displays four main bands, which can be associated to the A_1g_ vibration mode of the magnetite (at 668 cm^−1^) and three broad bands centered at 350 cm^−1^, 500 cm^−1^, and 700 cm^−1^, corresponding to the T_2g_, E_g_, and A_1g_ modes of maghemite, respectively [40,41,42]. Other characteristic features of the synthesized material, such as the black coloration of the powders and their strong interaction with external magnetic fields, were also observed. In the case of the rGO-based hybrids, the Raman spectrum displays the two characteristic bands of rGO, i.e., D and G at 1348 cm^−1^ and 1594 cm^−1^, respectively, along with the characteristic intensity band ca. 680 cm^−1^ associated with the A_1g_ modes of the Fe_3_O_4_ (668 cm^−1^) and γ-Fe_2_O_3_ (700 cm^−1^) magnetic phases.

### 3.2. Electrochemical Measurements

**Figure 3:**
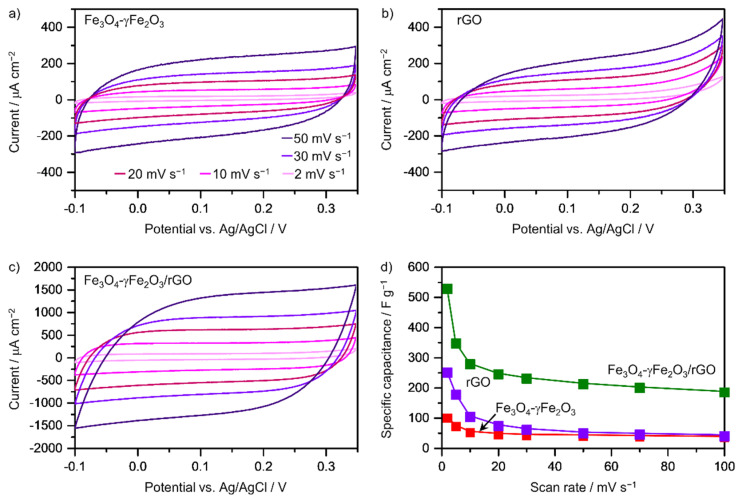
Cyclic voltammetries of (**a**) clusters of Fe_3_O_4_/γ-Fe_2_O_3_ nanoparticles, (**b**) rGO sheets and (**c**) rGO-Fe_3_O_4_/γ-Fe_2_O_3_ nanocomposite material at different scan rates. (**d**) Specific capacitance as a function of the scan rate for the three materials under study. Experimental conditions: 3 M KOH solution, room temperature.

The electrochemical performance of the samples was investigated by cyclic voltammetry, galvanostatic charge–discharge scanning experiments at different scan rates, and electrochemical impedance spectroscopy. Figure 3 shows the cyclic voltammetries obtained from −0.1 V to +0.35 V in 3 M KOH at different scan rates ranging from 2 mV s^−1^ to 50 mV s^−1^. The specific capacitance (*C_s_*) (F g^−1^) is calculated from the cyclic voltammetry curves using Equation (1) [36], in which  ΔI corresponds to the difference in the peak oxidation and reduction currents in amperes (A), *m* is the mass loading in grams (g), and υ is the scan rate, in V s^−1^.

(1) $$C_{s}=\frac{\Delta I}{m\upsilon }$$

The values obtained for a scan rate of 2 mV s^−1^ scan rate were 100 F g^−1^, 250 F g^−1^, and 528 F g^−1^ for the clusters of Fe_3_O_4_-γFe_2_O_3_ nanoparticles, the rGO sheets, and the Fe_3_O_4_-γFe_2_O_3_/rGO hybrid structure, respectively (Figure 3). Chen et al. reported a specific capacitance of 262.1 F g^−1^ for Fe_3_O_4_/rGO composites at the same scan rate and using 1 M Na_2_SO_3_ as the electrolyte [43], and Qi et al. reported a value of 350.6 F g^−1^ for Fe_3_O_4_/rGO composites at a scan rate of 1 mV s^−1^ and using 6 M KOH as the electrolyte [44]. On the other hand, Sheng et al. reported a graphene/Fe_3_O_4_ nanocomposite that exhibits a specific capacitance of 268 F·g^−1^ at 2 mV·s^−1^ using 1 M Na_2_SO_4_ as the electrolyte [25], and the fabrication of magnetite (Fe_3_O_4_)-decorated carbon nanotubes with a specific capacitance of 145.4 F g^−1^ at 2 mV·s^−1^ using 0.5 M Na_2_SO_4_ as electrolyte was reported by Nawwar et al. [11]. Additional data are included in Table S1 for comparative purposes. The energy density is 14.85 W h kg^−1^ at a power density of 1116.5 W kg^−1^, which is in line with previously reported results [15,45]. The clusters of iron oxide nanoparticles show the lowest specific capacitance of the three materials under study, which could be ascribed to the low conductivity of the iron oxide phases and/or poor electrolyte access to the surface of the particles. These aspects are improved in the case of rGO sheets, as it can be seen from the comparison of this material with the clusters of iron oxide nanoparticles. In the case of the hybrid structure, in which the clusters of iron oxide nanoparticles are interlayered in between the conductive rGO sheets, the specific surface area is expected to increase, thus increasing the electrolyte permeation through the rGO layers [31]. Figure 3d summarizes the specific capacitance values as a function of the scan rate. For a uniformly accessible surface, the capacitance should not depend on the scan rate, since the variations in the scan rates should result in equal magnitude variations of the difference in the peak oxidation and reduction currents given in Equation (1) [46]. However, the data presented in Figure 3d point to an unevenly accessible surface. The specific capacitance decreases exponentially with the increase in the scan rate, which points towards a decrease in the active surface. This can arise from the fact that, while at lower scan rates the electric field is able to penetrate the whole electrode structure, for higher scan rates only the outer part of the electrode material participates in the charging/discharging process [47].

**Figure 4:**
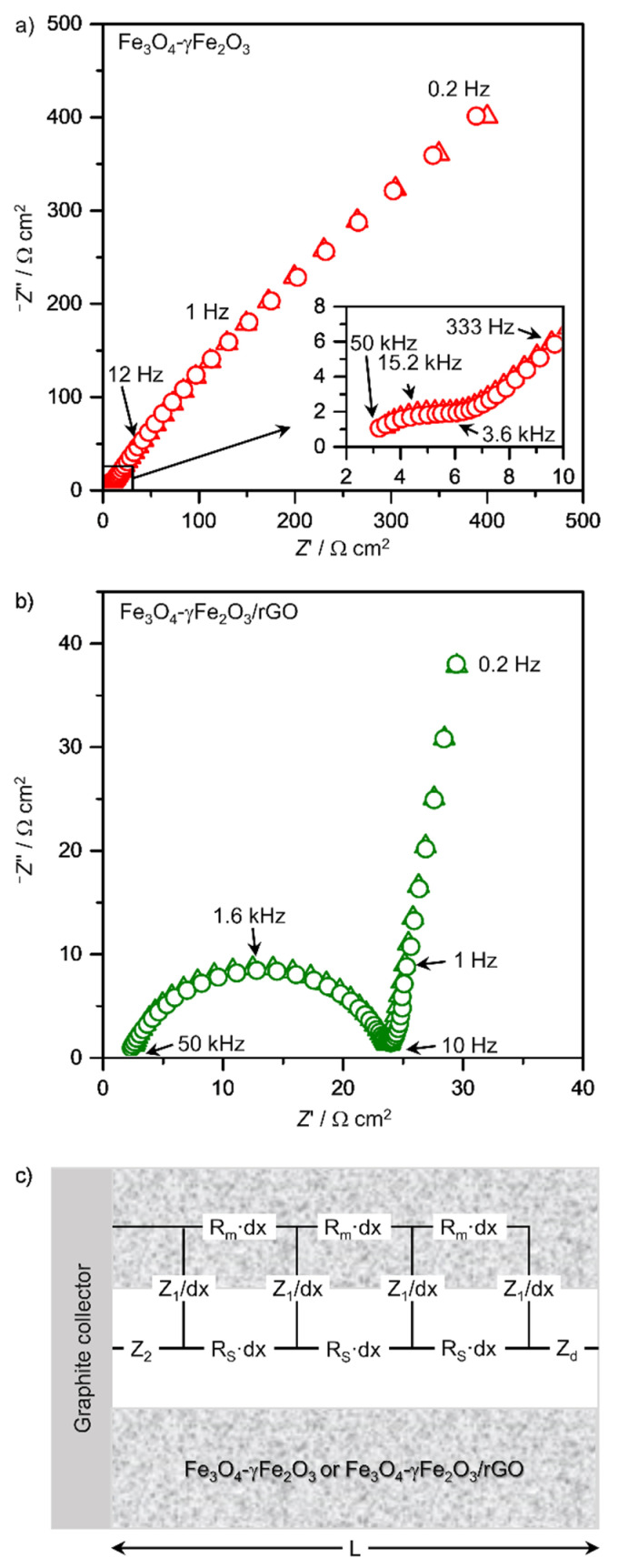
Nyquist impedance plots for (**a**) the clusters of Fe_3_O_4_-γFe_2_O_3_ nanoparticles and the control sample Fe_3_O_4_-γFe_2_O_3_/rGO-C and (**b**) Fe_3_O_4_-γFe_2_O_3_/rGO hybrid material. (**c**) Electrical equivalent circuit employed to model the experimental EIS data.

To confirm this hypothesis, EIS measurements were carried out on three selected systems: bare clusters of Fe_3_O_4_-γFe_2_O_3_ nanoparticles, the control sample Fe_3_O_4_-γFe_2_O_3_/rGO-C, and the Fe_3_O_4_-γFe_2_O_3_/rGO nanocomposite material. Figure 4 includes representative EIS Nyquist plots obtained for the systems tested, as well as the equivalent circuit employed for the data modeling. The Nyquist impedance plot of Fe_3_O_4_/γ-Fe_2_O_3_ clearly shows two domains in the low-frequency region, a capacitive arc section between 10 and 1 Hz, and a straight line with a 45° slope at the lowest frequencies. Moreover, the inset in Figure 4a, which corresponds to the high-frequency limit, clearly shows a depleted capacitive arc starting at about a 45° slope. This shape is characteristic of porous electrodes with de Levie type impedance behavior [48]. Figure 4b compiles the Nyquist impedance plot of the Fe_3_O_4_-γFe_2_O_3_/rGO hybrid material. Although the shape of the diagram is very different from that of the clusters of iron oxide nanoparticles, the same conclusions can be extracted if overlapping in the time constants in the high and medium frequency ranges is considered. The depletion of the high capacitive arc remains close to 45°. The porous structure for Fe_3_O_4_-based systems was described first for magnetite scales in heat exchangers [49], and later applied to other systems such as particulate zinc-rich coatings [50], conversion coatings for Li-ion batteries [51], or aged passive layers in concrete [52].

The low-frequency feature, i.e., the 45° tilt in the Nyquist plot of Figure 4a, is characteristic of a planar diffusion. The nanostructured electrodes behave in this way as flat electrodes due to the overlapping of the concentration profiles [53]. A relevant parameter for this research is the effect of rGO on the electrical conductivity of the nanostructured layer of particles. The EIS data can provide such information via a suitable electrical equivalent circuit that accounts for the physical phenomena above discussed.

Figure 4c includes a scheme of the electrical equivalent circuit employed to model the experimental EIS data. R_m_ accounts for the resistivity (electronic conduction) of the solid phase, i.e., the clusters of iron oxide nanoparticles or the nanocomposite material, and R_s_ corresponds to the resistivity (ionic conduction) associated with the electrolyte filling the layer pores. Z_1_ and Z_2_ account for the interfacial impedances at the pore wall and bottom, respectively, and Z_d_ accounts for the impedance associated with ionic diffusion to and from the electrode in the conditions of the quiescent solution employed.

The impedance functions Z_1_, Z_2_, and Z_d_ are defined as follows:

(2) $$Z_{1}\left(\omega \right)=\frac{R_{1}}{1+{j\omega R}_{1}C_{1}}\;(\mathrm{applies}\;\mathrm{to}\;{\mathrm{Fe}}_{3}O_{4}\text{-}\gamma {\mathrm{Fe}}_{2}O_{3}\;\mathrm{and}\;\gamma {\mathrm{Fe}}_{2}O_{3}/\mathrm{rGO}\text{-}C)$$

(3) $$Z_{1}\left(\omega \right)=R_{1}\left(1+{\left({j\omega R}_{1}C_{1}\right)}^{-\alpha_{1}}\right)\;(\mathrm{applies}\;\mathrm{to}\;{\mathrm{Fe}}_{3}O_{4}\text{-}\gamma {\mathrm{Fe}}_{2}O_{3}/\mathrm{rGO})$$

(4) $$Z_{2}\left(\omega \right)=\frac{R_{2}}{1+{\left({j\omega R}_{2}C_{2}\right)}^{\alpha_{2}}}$$

(5) $$Z_{d}\left(\omega \right)=R_{d}\frac{\sqrt{{j\omega \tau }_{d}}}{\tan h\sqrt{{j\omega \tau }_{d}}}$$

in which R_1_ and R_2_ correspond to the charge transfer resistances at the pore wall and bottom, respectively; C_1_ and C_2_ are the corresponding parallel double layer capacitances; R_d_ represents the diffusion resistance; τ_d_ is the time constant; $j=\sqrt{-1}$, and ω=2πf, in which f represents the frequency. α_2_ accounts for the Cole–Cole-type dispersion of the R_2_C_2_ time constant, which is associated to heterogeneities at the pores bottom. The R_1_C_1_ time constant in Equation (3) requires α_1_ to improve the fitting.

Equations (2) and (3) illustrate the remarkable difference between the clusters of iron oxide nanoparticles and the nanocomposite material. The former shows conductive behavior (Equation (2)), possibly ascribed to the redox transformation between Fe_3_O_4_ and γFe_2_O_3_ (2Fe_3_O_4_ + 2OH^−^ ↔ 3γFe_2_O_3_ + H_2_O + 2e^−^) [54]. In contrast, the hybrid exhibits blocking interfacial behavior (Equation (3)). The diffusion impedance defined in Equation (5) probably relates to the flow of OH^−^ species involved in the redox process and is not present in the hybrid structure, which is consistent with the blocking character of the pore walls that hinder the ionic flow.

The selected electrical equivalent model is able to reproduce accurately the experimental data, as shown in Figure 4a,b, which enables an in-depth analysis of the EIS spectra. The parameters compiled in Table 1 show that the only relevant difference between the bare Fe_3_O_4_-γFe_2_O_3_ nanostructure and Fe_3_O_4_-γFe_2_O_3_/rGO-C seems to be, as expected, the electronic conductivity of the materials, which increases by more than two orders of magnitude upon the introduction of rGO (R_m_ decreases from 11,200 to 23 Ω cm). This increase is accompanied by minor changes in the porosity of the pore network, as R_s_ increased only from 16 to 24 kΩ cm. The active surface increased by one order of magnitude, as indicated by the change in C_1_ from 7.1 to 63.1 mF cm^−3^. Concerning the low-frequency part of the spectra, the diffusion process from the electrolyte to the solid material, or vice versa, seems to slow down in the presence of rGO (τ_d_ increases from 0.23 to 17.7 s and R_d_ from 38.9 to 247.4 Ω cm^2^). This could be ascribed to the increased diffusion length due to the faster charge transfer associated with the higher active surface and higher conductivity of the Fe_3_O_4_-γFe_2_O_3_/rGO-C. Considering the thickness of the diffusion layer (δ), as $\delta =\sqrt{\tau D}$ and diffusivity D = 10 ^−5^ cm^2^ s^−1^, this parameter increases from 15 to 130 µm due to the presence of rGO.

In the case of the composite material, although the Nyquist impedance plot is different from that of either the clusters of iron oxide nanoparticles or the control sample Fe_3_O_4_-γFe_2_O_3_/rGO-C, the electrical equivalent model is very similar, indicating an analogous structure of the composite layer. The latter exhibits a higher C_1_ value associated to its blocking interfacial behavior. It also displays lower R_s_ and higher R_m_ values. In addition, the pores bottom is well differentiated from the pores wall, as typical double layer capacitance values in conducting interfaces, i.e., in the µF cm^−2^ range, are obtained for C_2_. It is also noticeable that the impedance spectra do not change significantly after cycling (data not shown), especially for the Fe_3_O_4_-γFe_2_O_3_/rGO hybrid nanocomposite, which is consistent with the performance upon prolonged cycling, as we will discuss later. It is important to note that the model applied in this study to interpret the impedance data (a transmission line model compatible with the material’s porous nature) is, to the best of our knowledge, new in the field, and presents a number of advantages: *(i)* it allows for accessing the resistivity of the active material (via R_m_) and its porosity (via R_s_); *(ii)* the kinetic information comes from R_1_ and R_2_, differentiating between charge transfer at the pore walls and at the pore bottom, respectively; and *(iii)* R_d_ accounts for the ionic flux from or towards the solution.

**Figure 5:**
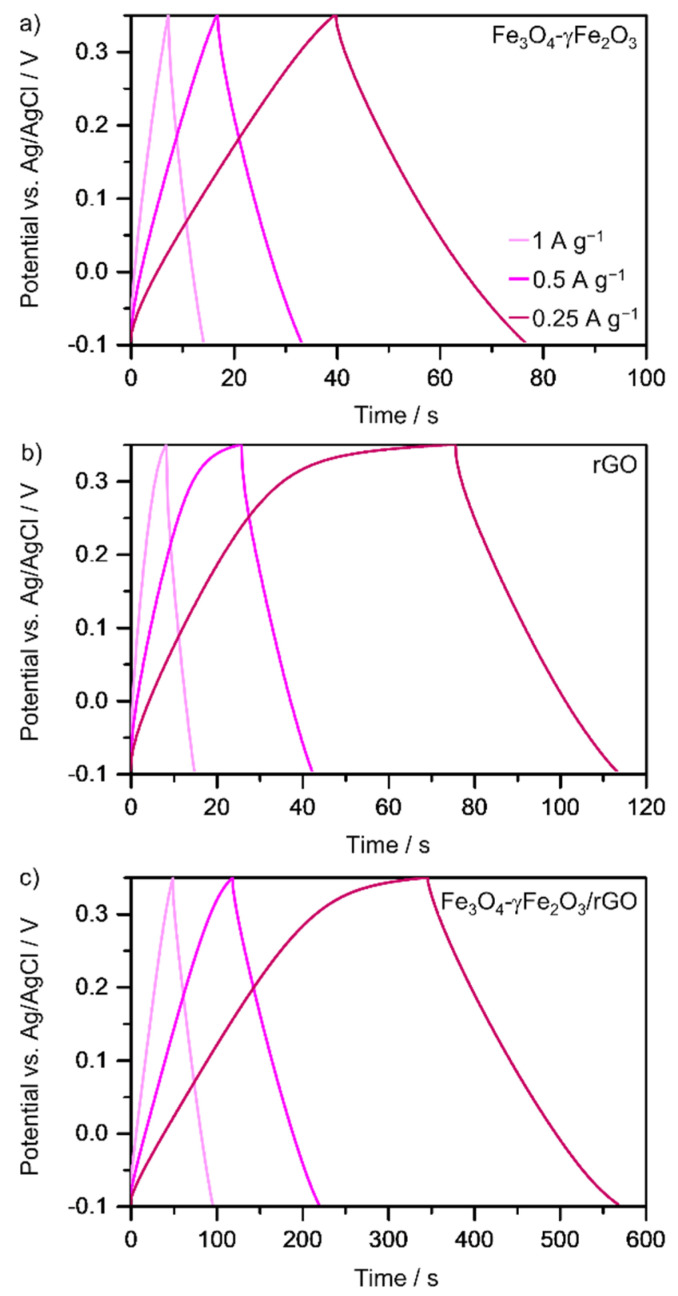
Galvanostatic charge–discharge curves of (**a**) the clusters of Fe_3_O_4_-γFe_2_O_3_ nanoparticles, (**b**) rGO, and (**c**) the Fe_3_O_4_-γFe_2_O_3_/rGO hybrid material at different current densities. Experimental conditions: 3 M KOH solution, room temperature.

The capacitive performance was also studied by galvanostatic charging–discharging at different current densities in the potential window from −0.1 to 0.35 V (Figure 5). As observed from Figure 5, the time for charging and discharging decreases as the current density increases. At higher current densities, the accessibility to the electrode porosity decreases because ions from the electrolyte do not have enough time to reach the pores bottom [55]. The charging–discharging time for the Fe_3_O_4_-γFe_2_O_3_/rGO nanocomposite material is much higher than that observed for both the clusters of iron oxide nanoparticles and rGO electrodes, which is in line with the higher capacitance value of the hybrid structure. As discussed above, the intimate interaction between the rGO substrate and the clusters of Fe_3_O_4_-γFe_2_O_3_ nanoparticles blocks the interface and enables facile electron transport, which is key to high specific capacitance and, consequently, a high charge–discharge time [28].

**Figure 6:**
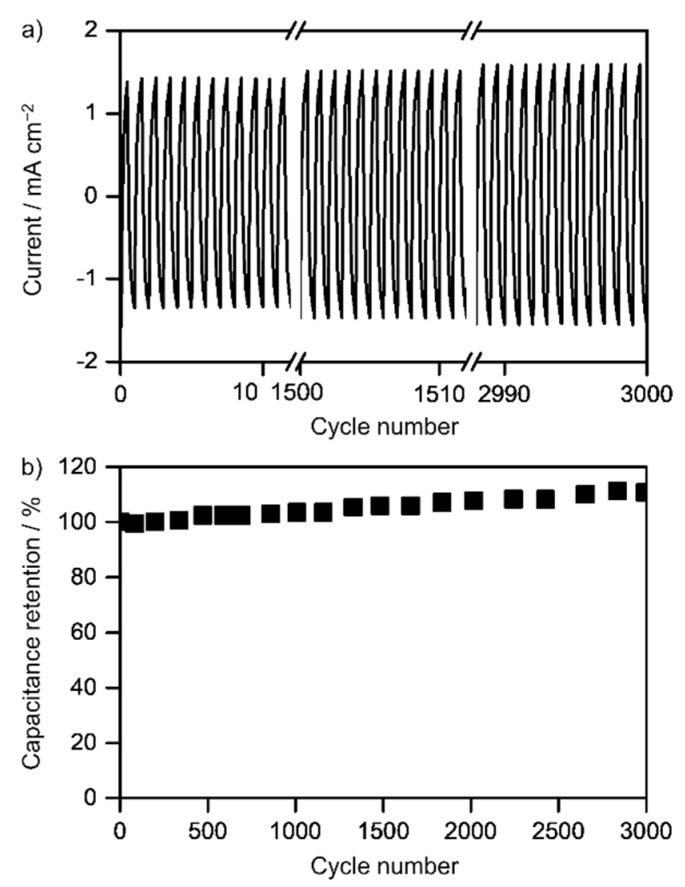
(**a**) Current and (**b**) capacitance retention of the Fe_3_O_4_-γFe_2_O_3_/rGO hybrid material as a function of the cycle number. Experimental conditions: 50 mV s^−1^, 3 M KOH solution, room temperature.

The long-term cycling stability of the Fe_3_O_4_-γFe_2_O_3_/rGO nanocomposite material was investigated for up to 3000 cycles on cyclic voltammetry at a scan rate of 50 mV s^−1^ in the potential window from −0.1 V to +0.35 V. The current evolution and coulombic efficiency as a function of the cycle number are summarized in Figure 6. It can be seen that the current (and hence the capacitance) increases with cycling. That increase is better visualized in the capacitance retention plot, which reaches 110% after 3000 cycles (Figure 6b). The growth of the clusters of iron oxide nanoparticles in between the rGO sheets avoids the aggregation of the oxidic phase, which, along with the improved dispersion of the clusters of Fe_3_O_4_-γFe_2_O_3_ nanoparticles owing to their interaction with the rGO substrate, results in the observed performance enhancement. Moreover, the large contact area between the active material and the electrolyte increases the efficiency of charge transport, thus also contributing to the capacitance improvement [56,57]. Although additional studies are needed in order to confirm these hypotheses, the obtained results highlight the potential of the fabricated hybrid nanocomposite to be employed as an electrode material for supercapacitor applications.

## 4. Conclusions

In this work, a composite material containing clusters of Fe_3_O_4_-γFe_2_O_3_ nanoparticles and reduced graphene oxide was developed through a simple one-step solvothermal synthesis method as a potential candidate for supercapacitors. The electrochemical study revealed that this hybrid structure, in which the clusters of iron oxide nanoparticles are interlayered in between the conductive rGO sheets, possesses much higher specific capacitance than the individual elements, used as reference. Specifically, 100 F g^−1^, 250 F g^−1^, and 528 F g^−1^ were obtained for the clusters of iron oxide nanoparticles, the rGO itself, and the composite material, respectively, at a scan rate of 2 mV s^−1^ using 3 M KOH as the electrolyte. In the case of the hybrid structure, the electrolyte permeation through the rGO layers increases, and consequently, so too does the capacitance. A state-of-the-art model was used to interpret the EIS measurements, enabling us to understand the reasons for the improved behavior, which was ascribed to the fact that the intimate interaction between the rGO substrate and the clusters of Fe_3_O_4_-γFe_2_O_3_ nanoparticles blocks the interface and enables facile electron transport. The evaluation of the performance of the nanocomposite material in subsequent cycles revealed that the current (and hence the capacitance) increases with the number of cycles, reaching 110% capacitance retention after 3000 cycles. The interaction of the clusters of iron oxide nanoparticles with the rGO substrate ensures a homogeneous dispersion of the oxidic phase embedded in between the rGO sheets, thus resulting in the observed capacitance enhancement. Although more detailed studies are needed in order to confirm these premises, the obtained insights pave the way towards the design of improved supercapacitors with superior performance.

## Figures and Tables

**Table 1:** Fitting parameter values obtained for the three systems investigated using the equivalent circuit depicted in Figure 4c. The thickness L of the nanostructured systems was fixed at 3.5 µm. R_0_ corresponds to the resistance of the electrolyte outside the sample, i.e., between the reference electrode and the surface of the sample, which depends on the position of the reference electrode with respect to the sample.

	R_0_/Ω cm^2^	R_m_/kΩ cm	R_s_/kΩ cm	R_1_/Ω cm^3^	C_1_/mF cm^−3^	α_2_	R_2_/kΩ cm^2^	C_2_/mF cm^2^	α_2_	R_d_/Ω cm^2^	τ_d_/s
Fe_3_O_4_-Fe_2_O_3_	0.2	11.2	16	1.1	7.1	-	20.8	9.3	0.639	38.9	0.23
Fe_3_O_4_-Fe_2_O_3_/rGO-C	1.8	0.023	24	1.6	63.1	-	20.6	15.0	0.657	247.4	17.7
Fe_3_O_4_-Fe_2_O_3_/rGO	0.1	5.5	6.2	7 × 10^−3^	56 × 10^3^	0.905	0.2	2 × 10^−3^	0.846	-	-

## Data Availability

Not applicable.

## Supplementary Materials

The following supporting information can be downloaded at: https://www.mdpi.com/article/10.3390/nano12152695/s1, Figure S1: TEM images of higher resolution of clusters of iron oxide nanoparticles, permitting to appreciate their aggregated nature; Table S1: Comparison of the performance of the Fe_3_O_4_-γFe_2_O_3_/rGO composite with state-of-the-art materials. References [11,15,17,25,43,44,58,59,60] are cited in the supplementary materials.
