# Supplementary material for: Synergistic Interaction of Clusters of Iron Oxide Nanoparticles and Reduced Graphene Oxide for High Supercapacitor Performance
Source: Nanomaterials (Basel). 2022 Aug 5;12(15):2695. doi: 10.3390/nano12152695 (PMC9370716; doi:10.3390/nano12152695)
Supplement: Supplementary file 1 [file nanomaterials-12-02695-s001.zip › nanomaterials-1792546-Supplementary.pdf]

Supplementary Materials

Synergistic Interaction of Clusters of Iron Oxide Nanoparticles and Reduced Graphene Oxide for High Supercapacitor Performance

Amir Elsaidy [1], Julia N. Majcherkiewicz [1,2], Begoña Puértolas [1,*], Verónica Salgueiriño [1,2,*], Xosé Ramón Nóvoa [3] and Miguel A. Correa-Duarte [1]

[1] CINBIO, Universidade de Vigo, 36310 Vigo, Spain;
[2] Departamento de Física Aplicada, Universidade de Vigo, 36310 Vigo, Spain
[3] CINTECX, ENCOMAT Group, EEI, Universidade de Vigo, 36310 Vigo, Spain;
* Correspondence: begona.puertolas@uvigo.es (B.P.); vsalgue@uvigo.es (V.S.)

a)

b)

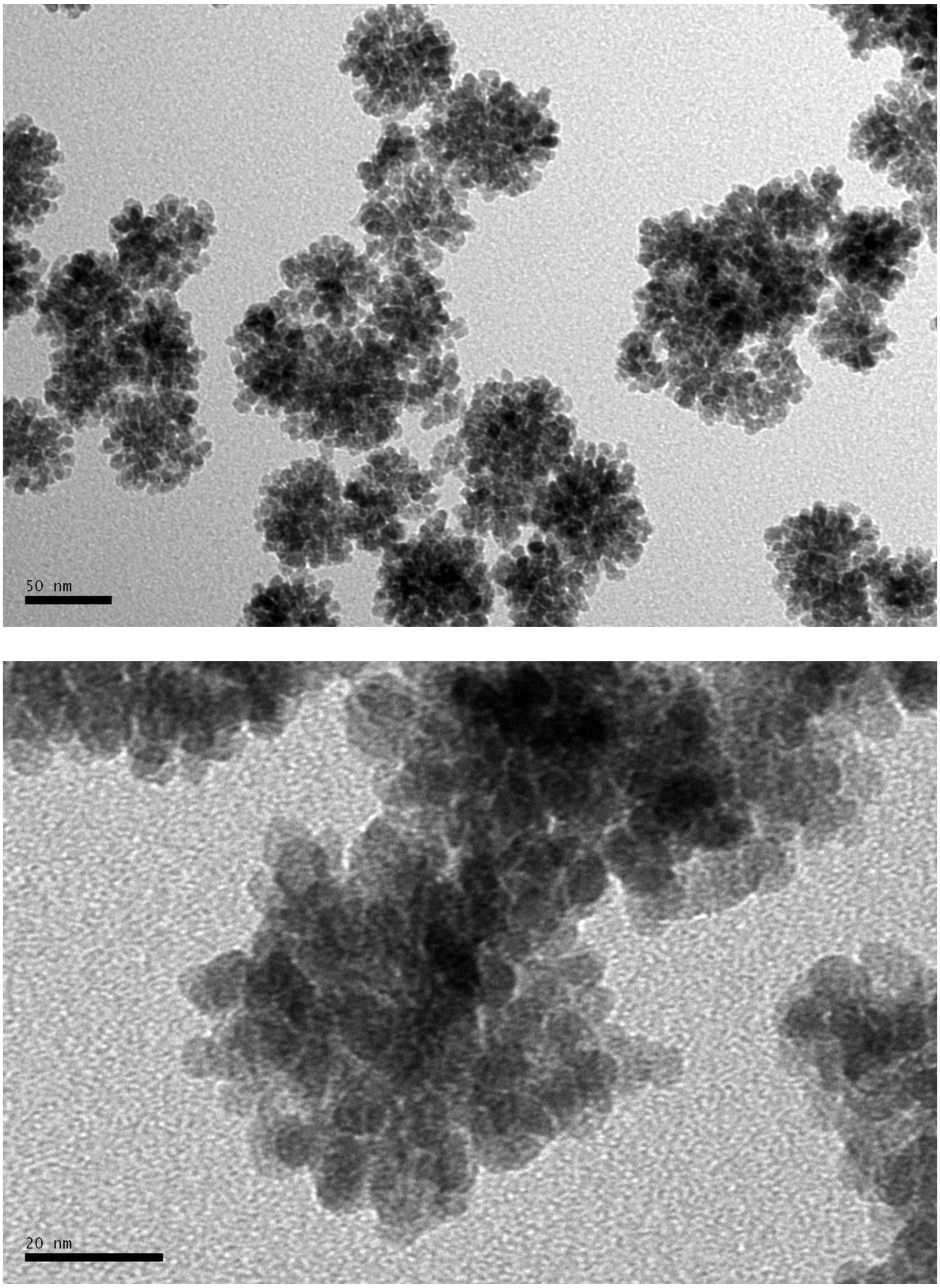

Figure S1. TEM images (**a**,**b**) of higher resolution of clusters of iron oxide nanoparticles, permitting to appreciate their aggregated nature.

Table S1. Comparison of the performance of the Fe_3O_4-γFe_2O_3/rGO composite with state-of-the-art materials.

**Material**	**Specific capacitance F g^{-1}**	**Scan rate mV s^{-1}**	**Electrolyte**	**Ref.**
Fe_3O_4-γFe_2O_3/rGO	528	2	3 M KOH	This work
Fe_3O_4/rGO	262.1	2	1 M Na_2SO_3	[1]
Fe_3O_4/rGO	350.6	1	6 M KOH	[2]
graphene/Fe_3O_4	268	2	1 M Na_2SO_4	[3]
Fe_3O_4-carbon nanotubes	145.4	2	0.5 M Na_2SO_4	[4]
Fe_2O_3 QDs/functionalized graphene oxide	347	10	1 M Na_2SO_4	[5]
Fe_3O_4@Fe_2O_3	231.9	5	1 M Na_2SO_4	[6]
Fe_2O_3@C	315	2	2 M KOH	[7]
Fe_2O_3/carbon black	40.07	10	2 M KCl	[8]
γ-Fe_2O_3/graphene	224	25	1 M Na_2SO_3	[9]

References

1. Cheng, J.; Shou, Q.; Wu, J.; Liu, F.; Dravid, V.P.; Zhang, X. Influence of component content on the capacitance of magnetite/reduced graphene oxide composite. *J. Electroanal. Chem.* **2013,** *698*, 1–8. https://doi.org/10.1016/j.jelechem.2013.03.017

2. Qi, T.; Jiang, J.; Chen, H.; Wan, H.; Miao, L.; Zhang, L. Synergistic effect of Fe_3O_4/reduced graphene oxide nanocomposites for supercapacitors with good cycling life. *Electrochimica Acta* **2013,** *114*, 674–680. https://doi.org/10.1016/j.electacta.2013.10.068

3. Sheng, S.; Liu, W.; Zhu, K.; Cheng, K.; Ye, K. Wang, G.; Cao, D.; Yan, J. Fe_3O_4 nanospheres in situ decorated graphene as high-performance anode for asymmetric supercapacitor with impressive energy density. *J. Colloid Interface Sci.* **2019**, *536*, 235–244. https://doi.org/10.1016/j.jcis.2018.10.060

4. Nawwar, M.; Poon, R.; Chen, R.; Sahu, R.P.; Puri, I.K.; Zhitomirsky, I. High areal capacitance of Fe_3O_4-decorated carbon nanotubes for supercapacitor electrodes. *Carbon Energy* **2019**, *1*, 124–133. https://doi.org/10.1002/cey2.6

5. Xia, H.; Hong, C.; Li, B.; Zhao, B.; Lin, Z.; Zheng, M.; Savilov, S.V.; Aldoshin, S.M. Facile synthesis of hematite quantum-dot/functionalized graphene-sheet composites as advanced anode materials for asymmetric supercapacitors. *Adv. Funct. Mater.* **2015**, *25*, 627–635. https://doi.org/10.1002/adfm.201403554

6. Tang, X.; Jia, R.; Zhai, T; Xia, H. Hierarchical Fe_3O_4@Fe_2O_3 core–shell nanorod arrays as high-performance anodes for asymmetric supercapacitors. *ACS Appl. Mater. Interfaces* **2015**, *7*, 27518-27525. https://doi.org/10.1021/acsami.5b09766

7. Sethuraman, B.; Purushothaman, K.K.; Muralidharan, G. Synthesis of mesh-like Fe_2O_3/C nanocomposite *via* greener route for high performance supercapacitors**.** *RSC Adv.* **2014**, *4*, 4631–4637. https://doi.org/10.1039/C3RA45025B

8. Nasibi, M.; Golozar, M.A.; Rashed, G. Nano iron oxide (Fe_2O_3)/carbon black electrodes for electrochemical capacitors. *Mater. Lett.* **2012**, *85*, 40–43. https://doi.org/10.1016/j.matlet.2012.06.109

9. Chen, H.-C.; Wang, C.-C.; Lu, S.-Y. γ-Fe_2O_3/graphene nanocomposites as a stable high performance anode material for neutral aqueous supercapacitors. *J. Mater. Chem. A* **2014**, *2*, 16955-16962. https://doi.org/10.1039/C4TA03574G
